# Circulating fibrocytes are not disease-specific prognosticators in idiopathic pulmonary fibrosis

**DOI:** 10.1183/13993003.00172-2021

**Published:** 2021-07-22

**Authors:** Iain D. Stewart, Henry Nanji, Grazziela Figueredo, William A. Fahy, Toby M. Maher, Antje J. Ask, Shyam Maharaj, Kjetil Ask, Martin Kolb, Gisli R. Jenkins

**Affiliations:** 1 Division of Respiratory Medicine, NIHR Biomedical Research Centre, University of Nottingham, Nottingham, UK; 2 Faculty of Medicine, National Heart and Lung Institute, Imperial College London, London, UK; 3 Advanced Data Analysis Centre, School of Computer Science, University of Nottingham, Nottingham, UK; 4 Discovery Medicine, GlaxoSmithKline R&D, GlaxoSmithKline Medicines Research Centre, Stevenage, UK; 5 National Institute of Health Research, Clinical Research Facility, Royal Brompton Hospital, London, UK; 6 Hastings Centre for Pulmonary Research and Division of Pulmonary, Critical Care and Sleep Medicine, Keck School of Medicine, University of Southern California, Los Angeles, CA, USA; 7 Division of Respirology, Dept of Medicine, McMaster University, Hamilton, ON, Canada; 8 Authors contributed equally to this manuscript

## Abstract

A number of previous studies have observed greater levels of circulating fibrocytes in interstitial lung disease compared to healthy controls, and suggest a prognostic role in idiopathic pulmonary fibrosis (IPF) [1–4]. Fibrocytes are circulating mesenchymal progenitor cells that differentiate into tissue specific fibroblasts and contribute to multiple wound healing processes, including secretion of inflammatory cytokines, contractile wound closure and promotion of angiogenesis [5]. However, the contribution of fibrocytes to the pathogenesis of progressive pulmonary fibrosis remains unclear and clinical observations require independent validation in prospective cohorts.

## Abstract 2

**In people with idiopathic pulmonary fibrosis, circulating fibrocytes ≥2.2% were associated with a greater risk of mortality over a median 3 years of follow-up, but were not associated with disease-related decline in lung function or short-term progression.** https://bit.ly/3bWydwQ

*To the Editor*:

A number of previous studies have observed greater levels of circulating fibrocytes in interstitial lung disease compared to healthy controls, and suggest a prognostic role in idiopathic pulmonary fibrosis (IPF) [1–4]. Fibrocytes are circulating mesenchymal progenitor cells that differentiate into tissue specific fibroblasts and contribute to multiple wound healing processes, including secretion of inflammatory cytokines, contractile wound closure and promotion of angiogenesis [5]. However, the contribution of fibrocytes to the pathogenesis of progressive pulmonary fibrosis remains unclear and clinical observations require independent validation in prospective cohorts.

Moeller *et al.* [1] observed that a 5% threshold of circulating fibrocytes could distinguish poor prognosis in a heterogeneous IPF cohort of 58 people, which included participants sampled during stability and at exacerbation. The PROFILE study is a prospective multicentre longitudinal cohort that includes patients diagnosed with IPF, confirmed using the same standards as Moeller *et al*. [1] and with circulating fibrocytes isolated and measured using identical protocols and settings. We tested whether the previously defined threshold of 5% in IPF was reliably associated with key clinical outcomes when sampled at stability with the goal of validating the role of circulating fibrocytes in informing IPF prognosis.

Fibrocytes were isolated and measured as described in Moeller *et al.* [1]. Briefly, the negative threshold for CD45 was set at 0.5%; cells gated for CD45 were analysed for collagen-1 expression. Specific staining for collagen-1 was determined as an increase in positive events over IgG isotype control thresholds set at 0.5%. The percentage of circulating fibrocytes from total circulating leukocytes were calculated for a cohort of 102 PROFILE participants, characterised by a median (interquartile range (IQR)) age of 73 (68–79) years, male predominance (74.5%) and mean forced vital capacity (FVC) and diffusing capacity of the lung for carbon monoxide (*D*_LCO_) of 82.8±20.7% predicted and 48.5±16.7% pred, respectively. We did not replicate a significant association with risk of death in unadjusted analysis (hazard ratio (HR) 1.57, 95% CI 0.83–2.98), nor when adjusted for baseline FVC and *D*_LCO_, age, sex, ever-smoker status and background therapy of steroid immunosuppression (HR 1.73, 95% CI 0.91–3.26) (figure 1a,b). Survival was censored at 1 March 2016; median (IQR) 38.1 (25.8–44.1) months follow-up.

**FIGURE 1:**
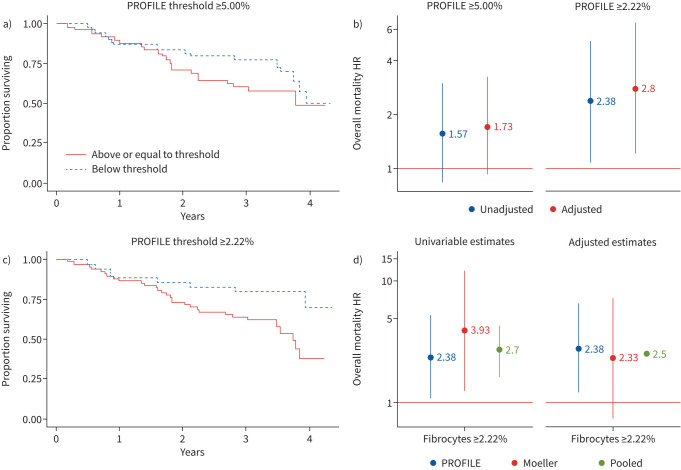
Circulating fibrocyte level and overall mortality in idiopathic pulmonary fibrosis (IPF). a) Kaplan–Meier survival curve at previously defined circulating fibrocyte threshold of 5% applied in PROFILE, log-rank test p=0.165; b) hazard ratio (HR) (95% CI) of circulating fibrocyte threshold in IPF; unadjusted and adjusted analyses in PROFILE at 5% and 2.22% threshold; c) Kaplan–Meier survival curve of circulating fibrocyte level at empirically defined threshold in PROFILE of 2.22%, log-rank test p=0.026; d) overall mortality at 2.22% threshold in unadjusted analysis and analysis adjusted for age, sex, baseline percentage predicted forced vital capacity and steroid immunosuppression estimated in PROFILE participants, Moeller *et al.* [1] participants and pooled in mixed-effect multilevel model.

It is possible that a greater frequency of acute exacerbations within the Moeller *et al*. [1] cohort created an effect size that was not reproducible in the PROFILE cohort, where fewer exacerbations were observed and samples were obtained at clinical stability. This suggests that circulating fibrocytes may be elevated only during exacerbations. A *post hoc* analysis of PROFILE data defined a Youden empirical cut-point during disease stability of 2.22% [6]. This threshold was significantly associated with unadjusted mortality in PROFILE (HR 2.38, 95% CI 1.09–5.21), as well as in the original derivation cohort (HR 3.93, 95% CI 1.27–12.21), and in a linear mixed-effect pooled analysis with random intercept at the study level (HR 2.70, 95% CI 1.65–4.42) (figure 1b–d). In adjusted analyses, circulating fibrocyte levels >2.22% were associated with a 2.8-fold (95% CI 1.21–6.49) greater risk of overall mortality in PROFILE and a 2.5-fold (95% CI 2.37–2.64) greater risk in the pooled analysis (figure 1d). These results confirm that higher levels of circulating fibrocytes can support clinical prognosis.

Moeller *et al*. [1] concluded that circulating fibrocytes predict mortality, but noted that significant differences were not observed between categories of clinical parameters that reflected disease severity, which included FVC, total lung capacity, *D*_LCO_ and 6-min walk test. The lack of an observed relationship with lung function or disease severity directly contrasts with other studies of IPF and other interstitial lung diseases, similarly limited by small numbers of participants [3, 4, 7]. We tested the association of circulating fibrocyte levels with baseline FVC % pred, *D*_LCO_ % pred and the dichotomised outcome of disease progression at 12 months defined by 10% relative decline in FVC or death. We observed no associations of circulating fibrocytes with FVC % pred (p=0.443), *D*_LCO_ % pred (p=0.423), nor with the composite disease progression end-point (p=0.447), confirming no observable effect in a large cohort of IPF at stability. These findings suggest that circulating fibrocytes are not a specific biomarker of fibroproliferation and functional decline. Ongoing efforts to characterise lung-specific fibrocytes may offer further insight into the prognostic potential of specific subtypes [2, 8].

The apparent discrepancy between the prognostic value of overall mortality and a lack of association with lung function or disease activity does not support circulating fibrocytes as a disease-specific biomarker of morbidity when assessed in IPF. Higher levels have been observed in individuals undergoing an acute exacerbation [1]; exacerbations may confound the prognostic potential of circulating fibrocytes relative to mortality. A limited number of healthy controls were also measured in the PROFILE cohort, with wide variability observed (IQR 0.63–4.66) that was not significantly different from IPF (IQR 1.22–8.80, p=0.086). This suggests that unrecorded morbidities in the healthy group, such as hypertension [9], may reflect elevated circulating fibrocytes in the absence of an interstitial lung disease.

This study has a number of limitations, including the small sample of healthy controls to detect differences between IPF and no IPF (n=11). 51 individuals with IPF had complete fibrocyte data at baseline and at 6 months, with no change over time detected (mean±sd difference 0.65±0.75%, p=0.38) and subsequent rationale for analysis of the first chronological instance of fibrocyte level in 102 individuals. Minimal change over time in IPF adds further support that circulating fibrocytes are not a useful metric of short-term fibrotic disease activity, while missingness reflects logistical difficulties in processing and measuring fibrocytes from clinical samples. Similar to Moeller *et al*. [1], the timing of recruitment meant that PROFILE participants were naïve to antifibrotics during the study and we could not evaluate circulating fibrocytes as a biomarker for clinical response to antifibrotic therapy, which may attenuate fibrocyte migration and fibroblast differentiation [10–12]. We did not perform sensitivity for IPF-related mortality; however, 84% (32 out of 38) of the PROFILE participants that died before censorship had pulmonary fibrosis recorded as a major cause of death. Major contributors to cause of death including ischaemic heart disease (seven out of 32), cancer (five out of 32), diabetes (four out of 32) and a range of vasculopathies were frequently recorded, including in the six individuals for whom pulmonary fibrosis was not recorded, yet the number of comorbidities recorded per participant was independent of their fibrocyte level (r_s_=0.109, p=0.240).

In summary, we observe that elevated circulating fibrocyte levels are associated with overall mortality in a large IPF cohort at stability, particularly at levels ≥2.2% of circulating leukocytes. Fibrocytes were not associated with IPF disease activity and elevated levels were not specific to disease status, suggesting that their value as a prognosticator is confounded by significant comorbidities and exacerbations commonly observed in IPF.

## Shareable PDF

10.1183/13993003.00172-2021.Shareable1 This one-page PDF can be shared freely online.
Shareable PDF ERJ-00172-2021.Shareable
