# Supplementary material for: Circulating fibrocytes are not disease-specific prognosticators in idiopathic pulmonary fibrosis
Source: Eur Respir J. 2021 Jul 22;58(1):2100172. doi: 10.1183/13993003.00172-2021 (PMC8295504; doi:10.1183/13993003.00172-2021)
Supplement: Supplementary file 1 [file ERJ-00172-2021.Shareable.pdf]

SHAREABLE PDF

Circulating fibrocytes are not disease-specific prognosticators in idiopathic pulmonary fibrosis

Iain D. Stewart [1,2], Henry Nanji [3], Grazziela Figueredo [3], William A. Fahy [4], Toby M. Maher [2,5,6], Antje J. Ask [7], Shyam Maharaj [7], Kjetil Ask [7], Martin Kolb [7,8] and Gisli R. Jenkins [1,2,8]

[1]Division of Respiratory Medicine, NIHR Biomedical Research Centre, University of Nottingham, Nottingham, UK. [2]Faculty of Medicine, National Heart and Lung Institute, Imperial College London, London, UK. [3]Advanced Data Analysis Centre, School of Computer Science, University of Nottingham, Nottingham, UK. [4]Discovery Medicine, GlaxoSmithKline R&D, GlaxoSmithKline Medicines Research Centre, Stevenage, UK. [5]National Institute of Health Research, Clinical Research Facility, Royal Brompton Hospital, London, UK. [6]Hastings Centre for Pulmonary Research and Division of Pulmonary, Critical Care and Sleep Medicine, Keck School of Medicine, University of Southern California, Los Angeles, CA, USA. [7]Division of Respirology, Dept of Medicine, McMaster University, Hamilton, ON, Canada. [8]Authors contributed equally to this manuscript.

Iain D. Stewart (iain.stewart@imperial.ac.uk)

Shareable abstract (@ERSpublications)
In people with idiopathic pulmonary fibrosis, circulating fibrocytes ⩾2.2% were associated with a greater risk of mortality over a median 3 years of follow-up, but were not associated with disease-related decline in lung function or short-term progression. https://bit.ly/3bWydwQ

Cite this article as: Stewart ID, Nanji H, Figueredo G, *et al*. Circulating fibrocytes are not disease-specific prognosticators in idiopathic pulmonary fibrosis. *Eur Respir J* 2021; 58: 2100172 [DOI: 10.1183/13993003.00172-2021].

This single-page version can be shared freely online.

Copyright ©The authors 2021.

This version is distributed under the terms of the Creative Commons Attribution Licence 4.0.

Received: 26 Sept 2020
Accepted: 11 March 2021

To the Editor:

A number of previous studies have observed greater levels of circulating fibrocytes in interstitial lung disease compared to healthy controls, and suggest a prognostic role in idiopathic pulmonary fibrosis (IPF) [1–4]. Fibrocytes are circulating mesenchymal progenitor cells that differentiate into tissue specific fibroblasts and contribute to multiple wound healing processes, including secretion of inflammatory cytokines, contractile wound closure and promotion of angiogenesis [5]. However, the contribution of fibrocytes to the pathogenesis of progressive pulmonary fibrosis remains unclear and clinical observations require independent validation in prospective cohorts.
